# Multiple Kinases Can Phosphorylate the N-Terminal Sequences of Mitochondrial Proteins in *Arabidopsis thaliana*

**DOI:** 10.3389/fpls.2018.00982

**Published:** 2018-07-10

**Authors:** Yee-Song Law, Ling Ngan, Junran Yan, Lok Y. Kwok, Yuzhe Sun, Shifeng Cheng, Serena Schwenkert, Boon L. Lim

**Affiliations:** ^1^School of Biological Sciences, The University of Hong Kong, Pokfulam, Hong Kong; ^2^Department of Biology I, Botany, Ludwig-Maximilians-Universität München, Munich, Germany; ^3^State Key Laboratory of Agrobiotechnology, The Chinese University of Hong Kong, Shatin, Hong Kong

**Keywords:** 14-3-3 protein, mitochondria, phosphorylation, presequence, protein import, STY kinase

## Abstract

Phosphorylation of the transit peptides of nuclear-encoded preprotein is a well-known regulatory process of protein import in plant chloroplasts. In the Arabidopsis Protein Phosphorylation Site Database, 103 out of 802 mitochondrial proteins were found to contain one or more experimentally proven phosphorylation sites in their first 60 amino acid residues. Analysis of the N-terminal sequences of selected mitochondrial preproteins and their homologs from 64 plant species showed high conservation among phosphorylation sites. The ability of kinases from various sources including leaf extract (LE), root extract (RE), wheat germ lysate (WGL), and STY kinases to phosphorylate N-terminal sequences of several respiratory chain proteins were examined by *in vitro* kinase assays. The three STY kinases were shown to phosphorylate the N-terminal sequences of some proteins we tested but exhibited different specificities. Interestingly, the N-terminal sequences of two mitochondrial ATP synthase beta subunit 1/3 (pF1β-1/3) could be phosphorylated by LE and RE but not by STY kinases, suggesting that there are uncharacterized presequence-phosphorylating kinases other than STY kinases present in RE and LE. Mitochondrial import studies showed that the import of RRL-synthesized pF1βs was impeded by the treatment of LE, and the addition of a short SSU transit peptide containing a phosphorylatable 14-3-3 binding site could enhance the import of LE-treated pF1βs. Our results suggested that the transit peptide of pSSU can compete with the presequences of pF1βs for an uncharacterized kinase(s) in leaf. Altogether, our data showed that phosphorylation of transit peptides/presequences are not uncommon for chloroplast-targeted and mitochondria-targeted proteins, albeit possibly differentially regulated.

## Introduction

Chloroplasts and mitochondria are known as “energy” organelles because they supply energy to cells. Chloroplasts produce ATP and reducing power through photosynthesis; whereas mitochondria produce ATP through respiration (Saraste, 1999; Eubel et al., 2004). Both “energy” organelles co-operate and inter-play to supply energy for plants in illuminated leaves (Yoshida et al., 2006). In Arabidopsis, nuclear-encoded chloroplast and mitochondrial preproteins are synthesized in the cytosol and post-translationally imported across the outer membranes of the chloroplasts and mitochondria through the translocases of the outer chloroplast (TOC) and mitochondrial (TOM) complexes (Schleiff and Becker, 2011). The TOM complex shares some common features with the TOC. For example, Tom20 functions like Toc34 in presequence/transit peptide recognition (Abe et al., 2000), whereas Tom40 and Toc75 are channels for translocation (Schleiff and Becker, 2011).

Several lines of evidence indicate that the phosphorylation state of chloroplast transit peptides regulates preprotein import (Martin et al., 2006; Lamberti et al., 2011b; Nickel et al., 2015). For example, the transit peptide of the precursor of the stroma localized small subunit of Rubisco (pSSU) is phosphorylated by three cytosolic plant-specific STY kinases and dephosphorylated by an unknown phosphatase before translocating into the TOC complex (Waegemann and Soll, 1996; Lamberti et al., 2011b). After phosphorylation at its transit peptide, pSSU forms a guidance complex with 14-3-3 protein and heat shock protein 70 (Hsp70), which is more import-competent than the unphosphorylated monomeric form of pSSU (May and Soll, 2000). In addition, mutations of the putative 14-3-3-binding sites on the transit peptides did not affect the import efficiency or fidelity *in vivo*, indicating that the 14-3-3 guidance complex system is dispensable (Nakrieko et al., 2004; Lee et al., 2006). Phosphomimicking within the transit peptide of pHCF136 and pSSU resulted in severe reduction of the import efficiency, suggesting that dephosphorylation is an obligatory prerequisite preceding translocation (Lamberti et al., 2011a; Nickel et al., 2015). The transit peptides of many chloroplast proteins were shown to bind to 14-3-3 and cytosolic Hsp70 (Fellerer et al., 2011). After translocation of the transit peptides, stromal Hsp70 (Chotewutmontri et al., 2012; Chotewutmontri and Bruce, 2015) and Hsp93 (Huang et al., 2016) can bind to the transit peptides, and hydrolysis of ATP by stromal Hsp70 will provide energy for protein import (Su and Li, 2010; Liu et al., 2014).

Until recently, phosphorylation and dephosphorylation of presequences of nuclear-encoded mitochondrial preproteins were not known to be involved in the import process (Murcha et al., 2014). However, the presequence of a nuclear-encoded mitochondrial preprotein, multiple organellar RNA editing factors 3 (MORF3), was recently shown to be phosphorylated by cytosolic STY kinases (Law et al., 2015). The import of pMORF3 synthesized in rabbit reticulocyte translation lysate (RRL-pMORF3) into mitochondria was impeded by phosphorylating its presequence by STY kinases, leaf extract (LE), and wheat germ translation lysate (WGL) (Law et al., 2015). In addition, the import rate of pMORF3 synthesized in WGL (WGL-pMORF3) into T-DNA insertional line of *Arabidopsis thaliana* purple acid phosphatase 2 (AtPAP2) was slower, but the import rate of the unphosphorylated RRL-pMORF3 was unaffected, suggesting that STY kinases and AtPAP2 might be involved in the import of pMORF3 into mitochondria by phosphorylating and dephosphorylating its presequences (Law et al., 2015). Like nuclear-encoded chloroplast preproteins, pMORF3 forms a complex with 14-3-3 proteins and Hsp70 after being phosphorylated by STY kinase. While the formation of 14-3-3/Hsp70 complexes speeds up the import of pSSU into chloroplasts (May and Soll, 2000), by contrast, formation of 14-3-3/Hsp70 complexes impedes the import of pMORF3 into mitochondria (Law et al., 2015).

Here, from the Arabidopsis Protein Phosphorylation Site Database (PhosPhAt 4.0) (Zulawski et al., 2013), we found that the N-terminal sequences of many nuclear-encoded mitochondrial preproteins contain experimentally determined phosphorylation sites. For verification, several nuclear-encoded preproteins of the respiratory chain were overexpressed and purified from *E. coli* and were shown to be phosphorylated by kinases from various sources such as LE, root extract (RE), WGL, and STY kinases. Therefore, phosphorylation of N-terminal sequences of nuclear-encoded mitochondrial preproteins is not uncommon in Arabidopsis. The N-terminal sequences of some mitochondrial preproteins can be phosphorylated by STY kinases (STY8, STY17, and STY46) but two homologs of precursor mitochondrial ATP synthase subunit beta 1/3 (pF1β-1/3) are not phosphorylated by STY kinases but by unknown protein kinases in the LE. Interestingly, RE can phosphorylate the N-terminal sequences of these three pF1β homologs, but not the other mitochondrial preproteins we tested, including pMORF3. Our data showed that uncharacterized kinases other than STY kinases might play similar biological roles in modulating protein translocation into mitochondria and different kinases would have different substrate specificity. Like what was observed with pMORF3, the import of RRL-synthesized pF1β is impeded by the treatment of LE, and the inclusion of a short SSU transit peptide (pSSU) containing a phosphorylatable 14-3-3 binding site could enhance the import of LE-treated pF1β, possibly by competing for kinase phosphorylation. The inclusion of a short SSU transit peptide (pSSU-M31/34-S/A) containing a non-phosphorylatable 14-3-3 binding site did not enhance pF1β import, suggesting that the transit peptide of pSSU can compete with the presequence of pF1β for an uncharacterized kinase(s) in leaf and thus phosphorylation and dephosphorylation of transit peptide/presequence are not uncommon for proteins imported into chloroplasts and mitochondria.

## Materials and Methods

### Computational Analyses

Nuclear-encoded mitochondrial preproteins extracted from two previous studies (Heazlewood et al., 2004; Duncan et al., 2011), were consolidated to create a single set of data. The first 60 amino acids of the N-terminal sequences of 802 nuclear-encoded mitochondrial preproteins were selected for phosphorylation motif analysis and 14-3-3 binding site prediction. 60 a.a. residues were used because a previous study on 62 Arabidopsis mitochondrial proteins reported that the average length of experimentally determined presequences was 50 a.a (Huang et al., 2009). For the post-translational modifications, “experimentally verified” and potential phosphorylation sites were extracted from PhosPhAt4.0 (Zulawski et al., 2013). Web server 14-3-3-Pred^1^ (Madeira et al., 2015) was used to predict the 14-3-3-binding sites on the N-terminal sequences of the mitochondrial proteins by combining predictions from three different classifiers: ANN – Artificial Neural Network (cut-off = 0.55), PSSM – Position-Specific Scoring Matrix (cut-off = 0.80), and SVM – Support Vector Machine (cut-off = 0.25). The final list of predicted phosphopeptides and 14-3-3 binding sites must fulfill the Consensus – Average of the scores provided by the three methods (cut-off = 0.50). For homologous protein searching, Blastp (e-value ≤1e-5, similarity ≥60%, aligned coverage ≥60%) was used to search for potential homologous proteins against the protein database of 64 species. Next, a HMM-based matrix (Hidden Markov Model) was built by HMMbuild to screen the identified homologs by HmmerSearch of the HMMER package (Eddy, 2011). After trimming, the first 70 amino acids of the retained homologous proteins were aligned by MUSCLE (Edgar, 2004). To create sequence logos, protein alignment was analyzed by Web server WebLogo^2^ (Crooks et al., 2004).

### Plasmid Construction

For overproduction of recombinant proteins in *E. coli*, first strand cDNA was reverse-transcribed by Reverse Transcription System (Promega, United States) according to the manufacturer’s instructions. The putative presequences of different precursor proteins were predicted from the WebLogo alignment, assuming that the mature sequences are highly conserved among the 64 species. Individual cDNA fragments encoding the 77 N-terminal residues of pF1β-1, 80 N-terminal residues of pF1β-2, 77 N-terminal residues of pF1β-3, 61 N-terminal residues of CAL2, 48 N-terminal residues of SDH1-2, 76 N-terminal residues of cytochrome C1, CytC1, 60 N-terminal residues of COX5b1, and 36 N-terminal residues of plant-specific subunit of the ATP17 were amplified by Platinum^®^ *Pfx* DNA Polymerase (Life Technologies, United States) using primers listed in Supplementary Table S6 and fused in frame with the pET21a vector (Novagen, Germany) containing Δ1-50aaMORF3 (Law et al., 2015). Construction of pET vectors for recombinant STY8, STY17, pMORF3, and Δ1-50aaMORF3 expression in *E. coli* was described previously Law et al. (2015). pTICHIS containing STY46 was obtained from Prof. Serena Schwenkert (Martin et al., 2006). To prepare vectors for *in vitro* mitochondrial import assay, the above pET21a vectors were used as templates for the generation of cDNAs containing *attB* sites and the PCR products were transferred into the pDONR201 vector (Life Technologies, United States) by Gateway BP reaction (Life Technologies, United States) according to the manufacturer’s instructions. The pDONR201 vector containing the fragment of interest was transferred into the pDEST14 vector (Life Technologies) by Gateway LR reaction (Life Technologies, United States) for *in vitro* protein translation.

### Plant Materials and Growth Conditions

For the mitochondrial import assay and preparation of RE, seeds of wild-type *Arabidopsis thaliana* ecotype Columbia-0 (WT) were sterilized with 20% (v/v) bleach and grown for 14 days on Murashige and Skoog (MS) medium supplemented with 3% (w/v) sucrose under long-day conditions [16 h light (22°C)/8 h dark (18°C)] in a controlled-environment chamber. For the preparation of LE, WT seeds were grown for 10 days on MS medium. Similar size of seedlings were transferred to soil and grown until 28 days under long-day conditions in a controlled-environment chamber.

### Preparation of Leaf Extract and Root Extract

Twenty-eight-day-old WT leaves and 14-day-old WT root were ground with mortar and pestle to powder in liquid nitrogen. Grinding buffer (20 mM Tris-HCl, pH 7.5, 10 mM β-mercaptoethanol) was added to the mortar and pestle and ground for 5 min. After that, the homogenate was filtered through four layers of cheesecloth and centrifuged at 350 × *g* for 10 min at 4°C. The supernatant was transferred to a new centrifuge tube and centrifuged at 12,000 × *g* for 20 min at 4°C. Next, the supernatant was further centrifuged at 100,000 × *g* for 1 h at 4°C by ultracentrifuge (Beckman). The final supernatant is the LE or RE.

### *In Vitro* Kinase Assay

pET expression vectors containing gene of interest were transformed into *E. coli* strain BL21 (DE3) pLysS. The overexpression and purification of recombinant proteins as well as *in vitro* kinase assay were performed as previously described by Martin et al. (2006) and Law et al. (2015). One microgram of recombinant substrate protein and kinase [recombinant STY8 (1 μg), recombinant STY17 (1 μg), recombinant STY46 (1 μg), LE (10 μg) and RE (10 μg)] were incubated at 30°C for 15 min in the reaction buffer (20 mM Tris-HCl, pH 7.5, 5 mM MgCl_2_, 0.5 mM MnCl_2_, 2.5 μM ATP) containing 5 μCi of [γ-^32^P] ATP. All the reaction was terminated by Lammeli sample buffer and analyzed by 15% (v/v) SDS-PAGE gel and exposed to a Hyperfilm MP (GE Healthcare, United States).

### Mitochondria Isolation

This method was previously performed by Day et al. (1985) and Lister et al. (2007). First, 14-day-old seedlings grown on MS agar were harvested and ground by mortar and pestle containing grinding buffer [300 mM sucrose, 25 mM tetrasodium pyrophosphate, 2 mM EDTA, 10 mM KH_2_PO_4_, 1.0% (w/v) PVP-40, 1.0% (w/v) BSA, pH 7.5] on ice. The ground sample was filtered by four layers of miracloth and centrifuged at 2,450 × *g* for 5 min at 4°C and followed by 17,500 × *g* for 20 min at 4°C. The supernatant was discarded and the pellet was gently resuspended in wash buffer [0.3 M sucrose, 20 mM TES, 0.2% (w/v) BSA, pH 7.5] and further centrifuged at 2,450 × *g* and 17,500 × *g* for 5 min and 20 min, respectively. Then, the crude mitochondria were resuspended in wash buffer and loaded onto Percoll^TM^ (GE Healthcare) containing 0–4.4% (w/v) PVP-40 gradient and centrifuged at 40,000 × *g* for 40 min at 4°C. A cloudy like band near the bottom of tube was transferred to new centrifuge tube and washed by wash buffer without BSA several times. Mitochondrial protein concentration was measured by Dye Reagent Concentrate Buffer (Bio-Rad).

### *In Vitro* Mitochondrial Protein Import

The *in vitro* mitochondrial protein import assay was performed as described previously Lister et al. (2007). T_N_T^®^ T7 Coupled Wheat Germ Extract System (Promega, United States) and T_N_T^®^ T7 Coupled Reticulocyte Lysate System (Promega, United States) were used to synthesize [^35^S]-Met-preproteins as described previously (Dessi et al., 2003; Lister et al., 2007). One import reaction contains 50 μg of mitochondria, 90 μl of ice-cold import master mix [300 mM sucrose, 50 mM KCl, 10 mM MOPS, 5 mM KH_2_PO_4_, 0.1% (w/v) mM BSA, 1 mM MgCl_2_, 1 mM methionine, 0.2 mM ADP, 0.75 mM ATP, 5 mM succinate, 5 mM DTT, 1 mM GTP, 1 mM NADH, pH 7.5] and 5 μl of [^35^S]Met-labeled preproteins either synthesized by T_N_T^®^ T7 Coupled Wheat Germ Extract System (Promega, United States) or T_N_T^®^ T7 Coupled Reticulocyte Lysate System (Promega, United States). For competition study, 5 μl of [^35^S] Met-labeled preproteins were pre-incubated with 5 μg LE and 0.5 mM synthetic peptide at 26°C for 2, 4 and 6 min in the reaction buffer (20 mM Tris-HCl, pH 7.5, 5 mM MgCl_2_, 0.5 mM MnCl_2_, 2.5 μM ATP) prior to performing the mitochondrial import assay. Phosphorylatable pSSU peptide (28-37; GLKSAASFPV) and non-phosphorylatable pSSU-M31/34-S/A peptide (28-37; GLKAAAAFPV) derived from tobacco pSSU were synthesized by Peptide 2.0 Inc (United States). Unimported preprotein was digested by 8 μl proteinase K (PK; 0.4 mg/μl), and the PK treatment was stopped by adding 1 μl of 100 mM PMSF. After discarding the supernatant, the mitochondrial pellet was resuspended in Lammeli sample buffer and denatured at 95°C for 5 min. The reaction products were separated by SDS-PAGE gel and detected by autoradiography.

### Co-immunoprecipitation Assay

[^35^S]Met-labeled preproteins synthesized in RRL (Promega, United States) were incubated with rotation for 1 h at RT in the presence of preimmune sera (GenScript, United States), anti-14-3-3 (a gift from Prof. Carol MacKintosh, University of Dundee) or anti-Hsp70 antibodies (Catalog no.: AS08 371; Agrisera, Sweden) in reaction buffer (50 mM Tris-HCl, 150 mM NaCl, 0.5 mM EDTA, 0.2% Triton X-100, pH 7.5) (Dessi et al., 2003). The antibodies were prepared against KLH-conjugated synthetic peptide conserved in the five cytosolic Hsp70 proteins of *Arabidopsis thaliana* (Sung et al., 2001). The bound proteins were pulled down using Protein-A Sepharose beads (Thermo Scientific, United States) and washed by reaction buffer containing 250 mM NaCl four times. After the supernatant was discarded, the Protein-A Sepharose beads (Invitrogen, United States) was resuspended in Lammeli sample buffer and denatured at 95°C for 5 min. The immunoprecipitated proteins were analyzed in SDS-PAGE gel and detected by autoradiography.

## Results

### Data-Mining of Experimentally Determined Phosphorylation Sites on the N-Terminal Sequences of Mitochondrial Preproteins

Based on a large-scale protein phosphorylation study of *Arabidopsis thaliana* (Wang et al., 2013), pMORF3 was revealed to contain three experimentally proven phosphorylation sites (Thr-17, Ser-20, and Thr-35) on its presequence. Furthermore, as evidenced by the *in vitro* kinase assay, cytosolic kinases STY8 and STY17 were shown to phosphorylate the presequences of pMORF3 (Law et al., 2015). To examine if the presequences of the other nuclear-encoded mitochondrial preproteins contain experimentally proven phosphorylation sites, the PhosPhAt 4.0 database (Zulawski et al., 2013) was utilized for data-mining experimentally determined phosphosites on the N-terminal sequences of mitochondrial preproteins. First, a total of 802 nuclear-encoded mitochondrial preproteins were extracted from two previous studies (Heazlewood et al., 2004; Duncan et al., 2011). The mitochondrial localization of these proteins was verified with the SUBA3 database (Tanz et al., 2013). From data mining of the PhosPhAt 4.0 Database, 103 out of the 802 nuclear-encoded mitochondrial preproteins were found to contain one or more experimentally proven phosphorylation sites on their first 60 amino acid residues (Supplementary Table S1). These phosphorylation sites could only be found on the N-terminal sequences of proteins targeted to the matrix or to the inner membrane, but not on the N-terminal sequences of the 64 proteins targeted to the outer membrane (Duncan et al., 2011). Of these 103 proteins containing phosphorylation sites in their N-terminal sequence, nineteen proteins are subunits of the mitochondrial respiratory complexes I–V (Supplementary Table S2) and the others play roles in TCA cycle, protein translation and degradation, RNA editing, transport, photorespiration, redox reactions, etc. In addition, more than 600 mitochondrial preproteins were predicted to contain one or more putative phosphorylation sites on their N-terminal sequences. In order to examine if phosphorylation at the N-terminal sequence is a common phenomenon, several protein subunits of respiratory complexes I–V, including CAL2, SDH1-2, COX5b1, ATP17 and F1β-1, F1β-2, F1β-3, were chosen for further experiments (**Table 1**).

**Table 1:** A list of the experimentally proven phosphorylation sites on the N-terminal sequences of some mitochondrial proteins examined in this study.

Name of protein	AGI code	Experimentally determined phosphorylation site	Reference
F1β-1	At5g08670	Ser54, Ser58, Ser61: VAEYAT(pS)SPA(pS)SAAP(pS)SAPAKDEGKK	Huang et al., 2009; Wang et al., 2013
F1β-2	At5g08680	Ser3: A(pS)RRILSSLLR Ser16: SSS(pS)RSTSKSSLIGSR	Wu et al., 2013; Zulawski et al., 2013
F1β-3	At5g08690	Ser31: LP(pS)PSPAR	Zhang et al., 2013
CAL2	At3g48680	Ser48: SQVTP(pS)PDR	Huang et al., 2009; Roitinger et al., 2015
SDH1-2	At2g18450	Thr22, Ser23, Ser26: SESNGAFI(pT)(pS)QL(pS)R	Zulawski et al., 2013
COX5b1	At3g15640	Ser21: TLAADVVAA(pS)PR Ser56: RF(pS)SDSVETPATK	Huang et al., 2009; Wang et al., 2013; Roitinger et al., 2015
ATP17	At4g30010	Ser8: VY(pS)EIRGK	Reiland et al., 2009; Reiland et al., 2011


**FIGURE 1:**
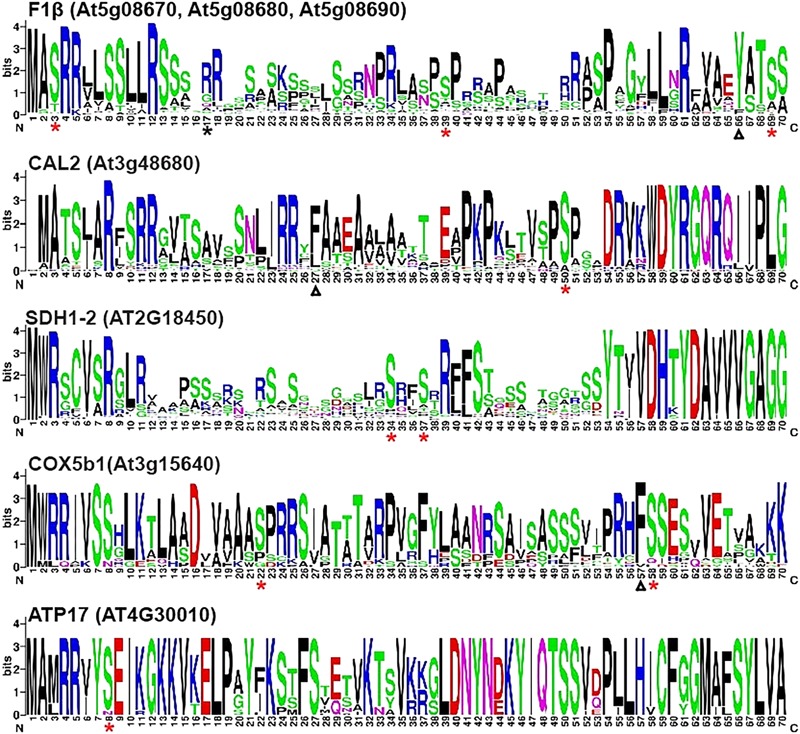
WebLogo presentation of N-terminal sequences of nuclear-encoded mitochondrial preproteins. Multiple alignment of the first 70 a.a. of homologous proteins from 64 plant species were analyzed by WebLogo (http://weblogo.berkeley.edu/logo.cgi) (Crooks et al., 2004). Asterisks represent experimentally proven phosphorylation sites. Red and black asterisks indicate the sites that are highly conserved and not conversed in the 64 plant species, respectively. The experimentally determined cleavage sites of the presequences of pF1β-1, CAL2, and COX5b1 were indicated by triangles (Huang et al., 2009).

A web server WebLogo (see footnote 2) (Crooks et al., 2004) was used to identify conserved phosphorylation sites among homologous proteins. Mitochondrial proteins listed in **Table 1** were used to search for homologous sequences from the protein databases of 64 plant species (Supplementary Table S3). The N-terminal sequences of the homologous proteins were aligned and analyzed by WebLogo to create sequence logos. The results of WebLogo may help us to predict the boundaries between the presequences and the mature proteins. For example, the protein sequences of SDH1-2 are highly conserved among 64 plant species after a.a. residue 52, implying that the mature protein sequences may start from this residue (**Figure 1**). As shown in **Figure 1**, the red asterisks on sequence logos represented the positions of the experimentally proven phosphorylation sites. These results suggested that the majority of the phosphorylation sites are conserved among their homologous proteins in the 64 plant species we examined. The only phosphorylation site presented in **Figure 1** that is not conserved is the Ser17 residue on the presequence of pF1β-2. This phosphorylation site is only found on pF1β-2 but not on pF1β-1/3 A study of 62 Arabidopsis, 52 rice, and 114 yeast mitochondrial proteins identified a conserved cleavage motif RX(F/Y/L)ˆ(S/A)(S/T) (Huang et al., 2009). The lengths of the presequences of pF1β-1, CAL2, and COX5b1 had been experimentally determined (Huang et al., 2009). Although two phosphorylation sites of pF1β-1 and COX5b1 are not located on their presequences, they are located on or near the cleavage motif (**Figure 1**).

### Prediction of Potential 14-3-3 Binding Phosphosites on the N-Terminal Sequences of Mitochondrial Proteins

Together with Hsp70, 14-3-3 proteins could bind to phosphorylated presequence of pMORF3 to form a complex (Law et al., 2015). In this study, a web server (Madeira et al., 2015) was employed to predict putative 14-3-3 binding phosphosites on the N-terminal sequences of nuclear-encoded mitochondrial preproteins. One hundred and ninety-eight out of the 802 mitochondrial proteins we examined were predicted to carry 260 putative 14-3-3 binding sites on their N-terminal sequences that fulfill the cut-offs of three different classifiers: ANN – Artificial Neural Network (cut-off = 0.55), PSSM – Position-Specific Scoring Matrix (cut-off = 0.80), and SVM – Support Vector Machine (cut-off = 0.25) (Supplementary Table S4). As shown in Supplementary Table S5, F1β-1, F1β-2 and F1β-3, CAL2, SDH1-2 and COX5b1 were predicted to carry one or more putative 14-3-3 binding phosphosites on their N-terminal sequences. However, no 14-3-3 binding phosphorylation site was predicted on the N-terminal sequences of ATP17 (Supplementary Table S5).

### STY8, STY17, and STY46 Have Different Substrate Specificities

**FIGURE 2:**
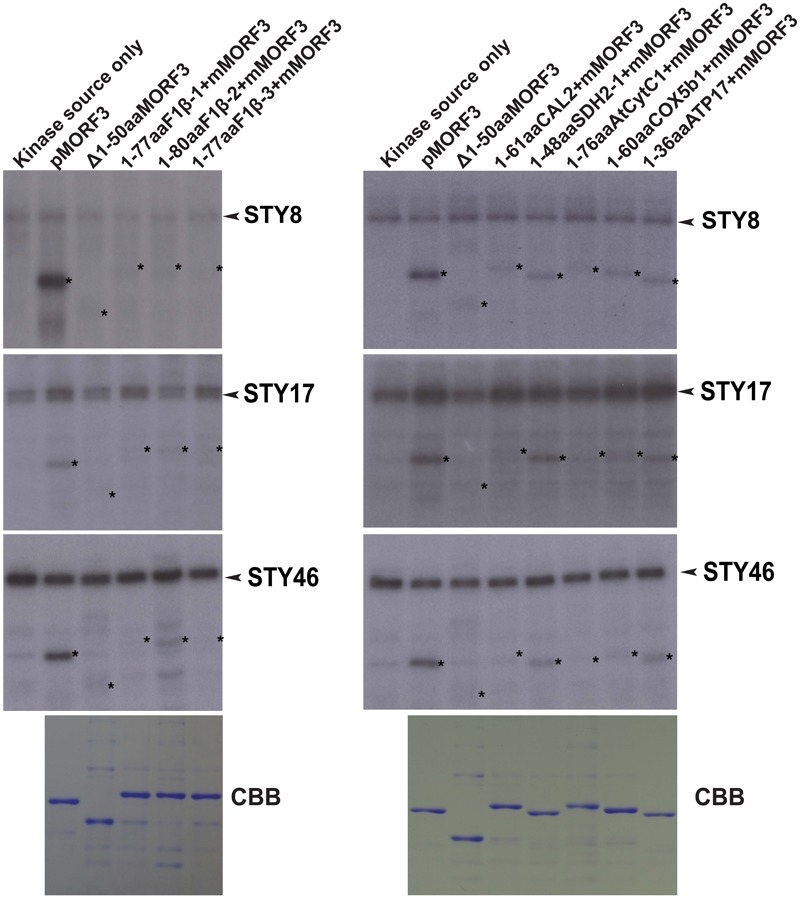
Phosphorylation of recombinant mitochondrial preproteins by STY kinases. Recombinant proteins with N-terminal sequences of selected mitochondrial proteins fused with the Δ1-50aaMORF3 were expressed in *E. coli* and purified. The abilities of recombinant STY8/17/46 kinase (1 μg) to phosphorylate various recombinant proteins were examined. CBB represents Coomassie Brilliant Blue staining. pMORF3 was used as a positive control; presequence deletion mutant of pMORF3 (Δ1-50aaMORF3) was used as a negative control. The N-terminal 77 residues of F1β-1, 80 residues of F1β-2, 77 residues of F1β-3, 61 residues of CAL2, 48 residues of SDH1-2, 76 residues of AtCytc1, 60 residues of COX5b1, and 36 residues of plant specific ATP17 were fused with Δ1-50aaMORF3 to create fusion proteins 1-77aaF1β-1 + Δ1-50aaMORF3, 1-80aaF1β-2 + Δ1-50aaMORF3, 1-77aaF1β-3 + Δ1-50aaMORF3, 1-61aaCAL2 + Δ1-50aaMORF3, 1-48aaSDH1-2 + Δ1-50aaMORF3, 1-76aaAtCytC1 + Δ1-50aaMORF3, 1-60aaCOX5b1 + Δ1-50aaMORF3 and 1-36aaATP17 + Δ1-50aaMORF3, respectively. The positions of the substrate proteins are indicated by asterisks.

The STY kinases were the first kinases reported to phosphorylate transit peptides of some nuclear-encoded chloroplast preproteins (Martin et al., 2006; Lamberti et al., 2011b). Interestingly, Law et al. (2015) showed that STY kinases could also phosphorylate the presequences of some mitochondrial preproteins. Here, we investigated whether N-terminal sequences of mitochondrial proteins listed in **Table 1** could be phosphorylated by STY kinases. In this experiment, pMORF3 was used as a positive control; presequence deletion mutant of pMORF3 (Δ1-50aaMORF3) was used as negative controls. First, recombinant proteins with the N-terminal sequences of selected mitochondrial proteins fused with the truncated MORF3 protein (Δ1-50aaMORF3) were expressed and purified from *E. coli* (**Figure 2**). By the *in vitro* kinase assay, STY8 was shown to phosphorylate the N-terminal sequences of SDH1-2 (1-48 a.a.), COX5b1 (1-60 a.a.) and ATP17 (1-36 a.a.) (**Figure 2**), whereas STY17 and STY46 phosphorylated only SDH1-2 (1-48 a.a.) and ATP17 (1-36 a.a.) (**Figure 2**). Interestingly, STY46 could also weakly phosphorylate F1β-2 (1-80 a.a.). The phosphorylation of these N-terminal sequences was generally weaker than that of pMORF3, which has three phosphorylation sites (T17/S20/T35) on its presequence. These results suggested that STY8, STY17, and STY46 have different but overlapping substrate specificities. These results corroborate with the different growth phenotypes of their T-DNA lines (Lamberti et al., 2011b).

### Kinases Properties in Leaf Extract and Root Extract Are Different

**FIGURE 3:**
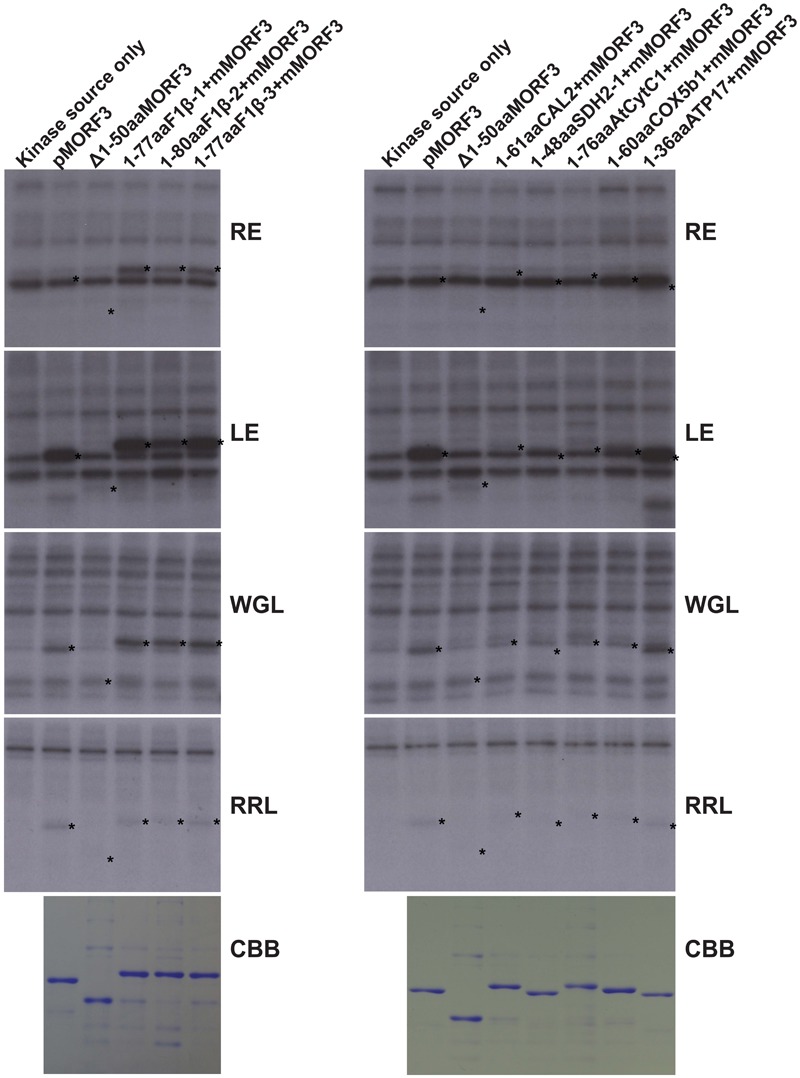
Phosphorylation of recombinant mitochondrial preproteins by various kinase sources. The abilities of LE (10 μg), RE (10 μg), WGL (10 μg, Promega), and RRL (65 μg, Promega) to phosphorylate various recombinant proteins (1 μg) were examined. CBB represents Coomassie Brilliant Blue staining. The positions of the substrate proteins are indicated by asterisks.

WGL and LE were previously reported to contain kinases that phosphorylate pMORF3 in the *in vitro* kinase assay (Law et al., 2015). However, the phosphorylating ability of kinases in RE was yet to be tested. The abilities of WGL (1 μl, Promega), RRL (1 μl, Promega), LE (10 μg), and RE (10 μg) to phosphorylate various recombinant proteins were examined in this study. We found that the kinases from RE could only phosphorylate the N-terminal sequences of the three pF1β homologous proteins, F1β-1 (1-77 a.a.), F1β-2 (1-80 a.a.) and F1β-3 (1-77 a.a.), but not the other mitochondrial proteins tested in this study, including pMORF3 (**Figure 3**). Furthermore, the N-terminal sequences of these three pF1β homologous proteins and ATP17 (1-36 a.a.) could also be phosphorylated by LE and WGL (10 μg) but only weakly by RRL (65 μg) (**Figure 3**). In summary, N-terminal sequences of F1β-1 and F1β-3 were not phosphorylated by STY kinases but could be phosphorylated by unknown kinases in LE and RE, indicating that uncharacterized kinases other than STY kinases could have different substrate specificities and various presequence-phosphorylating kinases might play similar biological roles in modulating protein translocation into mitochondria. Recombinant SDH1-2 protein can be phosphorylated by 1 μg STY kinases (**Figure 2**) but not by 10 μg LE or RE (**Figure 3**), possibly because the amounts of STY kinases in LE or RE is not sufficient (much lower than 1 μg) to produce intense signals like pMORF3 and ATP17 in 20 min. Alternatively, STY kinases maybe inhibited by inhibitors in LE and RE. The uncharacterized kinases other than STY kinases in LE or RE may be very active in phosphorylating the N-terminal sequences of pF1βs, pMORF3 and ATP17, due to a higher abundance in LE or RE, and/or a higher specific enzyme activity.

### pF1β-1 Forms Complex With Hsp70 and 14-3-3 After LE Treatment

**FIGURE 4:**
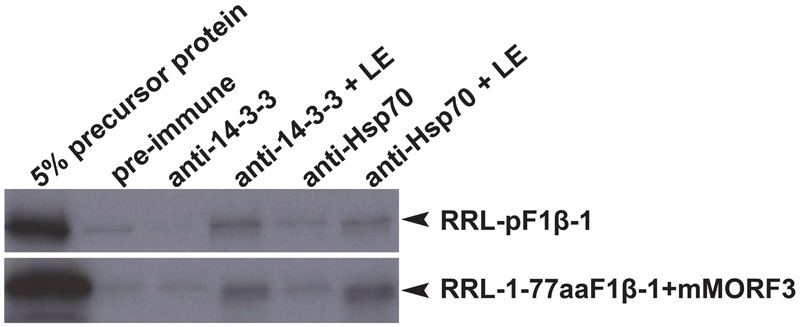
Co-immunoprecipitation assays of pF1β-1. The binding of RRL-pF1β-1 and RRL-1-77aaF1β-1 + mMORF3 to Hsp70 and 14-3-3 were strongly enhanced by LE treatment.

In co-immunoprecipitation assays, the phosphorylated form of pSSU and pMORF3 could form a guidance complex with 14-3-3 proteins and Hsp70 (May and Soll, 2000; Law et al., 2015). As LE but not RRL strongly phosphorylated N-terminal sequences (1-77/80 residues) of F1β-1/2/3 (**Figure 3**), we tested whether pF1β-1 synthesized in RRL (RRL-pF1βs) could interact with Hsp70 and 14-3-3 before and after LE treatment by a co-immunoprecipitation assay. As shown in **Figure 4**, RRL-pF1β-1 could only bind to Hsp70 and 14-3-3 after LE treatment. In order to test if the first 77 residues of F1β-1 could bind to Hsp70 and 14-3-3, the N-terminal sequence of pF1β-1 was fused to a truncated MORF3 protein (1-77aaF1β-1 + mMORF3) and the fusion protein was synthesized in RRL. The fusion protein could only bind to Hsp70 and 14-3-3 after LE treatment (**Figure 4**). Since RRL-pMORF3-T17/S20/T35A does not bind to Hsp70 and 14-3-3 even after kinase treatment (Law et al., 2015), the first 77 residues of F1β-1 must contain Hsp70 and 14-3-3 binding sites, and the binding is enhanced by LE treatment.

### pSSU Transit Peptide Can Compete for Kinase Phosphorylation and Enhance Import of pF1β Into Mitochondria

**FIGURE 5:**
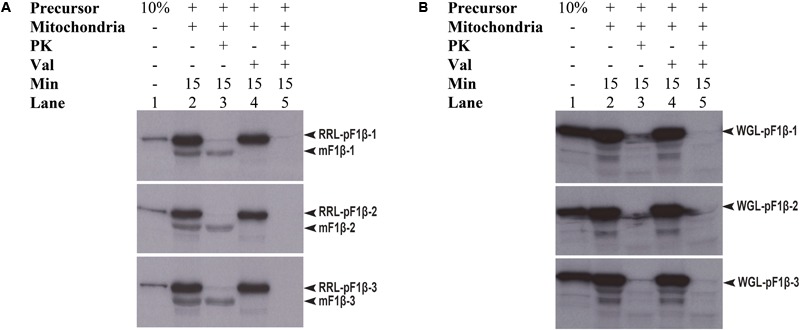
Mitochondrial import efficiency of RRL-pF1β-1/2/3 and WGL-pF1β-1/2/3 under various conditions. **(A)** RRL-pF1β-1/2/3 were readily imported into mitochondria (lanes 2 and 3) and the import was abolished in the presence of valinomycin (lanes 4 and 5). Proteinase K (PK) was used to digest non-imported RRL-pF1β-1/2/3 and WGL-pF1β-1/2/3 (lanes 3 and 5). **(B)** WGL-pF1β-1/2/3 were not import competent.

Preproteins synthesized in WGL have in some cases been reported to be import-incompetent into mitochondria, such as WGL-synthesized precursor *Nicotiana plumbaginifolia* F1β (NpF1β) (Dessi et al., 2003). However, Law et al. (2015) showed that pMORF3 synthesized in WGL (WGL-pMORF3) could be competently imported into *Arabidopsis thaliana* mitochondria but its import rate is far slower than that of RRL-pMORF3. The protein sequences of pF1β-1/2/3 (**Table 1**) share more than 97% sequence identity with each other and share about 82% a.a. sequence identity with NpF1β (Supplementary Figure S1). Since the differential phosphorylation in WGL and RLL may contribute to the differential inhibition of import, we investigated whether precursor F1β-1/2/3 synthesized in RRL (RRL-pF1β-1/2/3) and WGL (WGL-pF1β-1/2/3) are import competent into *Arabidopsis thaliana* mitochondria. As shown in **Figure 5**, RRL-pF1β-1/2/3 but not WGL-pF1β-1/2/3 were imported into Arabidopsis mitochondria.

**FIGURE 6:**
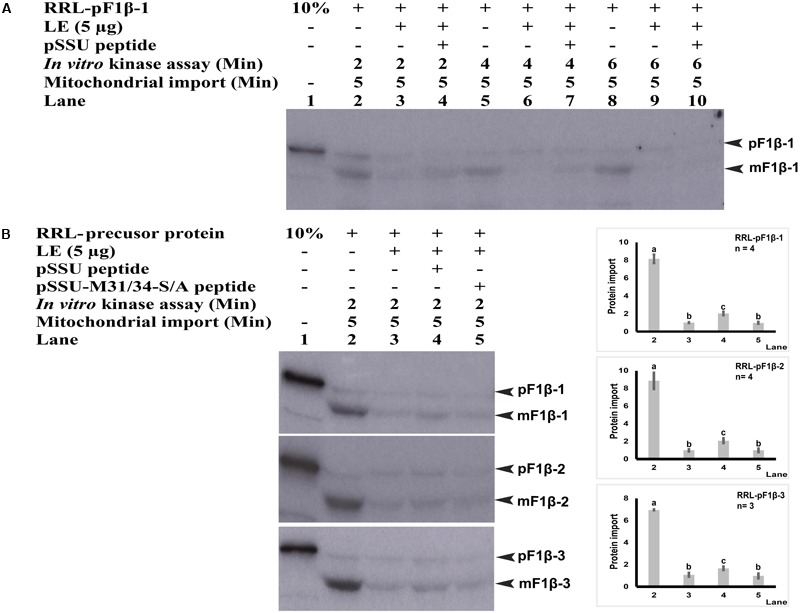
Mitochondrial import efficiency of RRL-pF1β-1/2/3 in the presence of pSSU and pSSU-M31/34-S/A peptides. **(A)** Pre-incubation of RRL-pF1β-1 with 5 μg LE (lanes 3 and 4, 6 and 7, 9 and 10), 0.5 mM pSSU peptides (lanes 4, 7, and 10) at 26°C for 2, 4, and 6 min. The import of LE-treated RRL-pF1β-1 (lanes 4 and 7) was enhanced by incubation with the pSSU peptide (lanes 3 and 6). However, when LE-treatment was extended 6 min, the SSU peptide had no effects. **(B)** RRL-pF1β-1/2/3 were pre-incubated with 5 μg LE (lanes 3–5), 0.5 mM pSSU peptides (lane 4) or non-phosphorylatable mutant pSSU-M31/34-S/A peptide (lane 5) at 26°C for 2 min. The import of LE-treated RRL-pF1β-1/2/3 was enhanced by the pSSU peptide (lane 4) by not by the pSSU-M31/34-S/A peptide (lanes 3 and 5). Import experiments were repeated three times and statistical differences were based on one-way ANOVA analysis followed by Tukey Honestly Significant Differences test. The amount of imported mature protein (lane 3) was taken as 1.0. The values marked with different letters (a, b, c) are significantly different (*P* < 0.05).

We have previously shown that LE and WGL could strongly phosphorylate pF1βs (**Figure 3**). LE and WGL were also shown to phosphorylate chloroplast transit peptides, including tobacco pSSU (Martin et al., 2006; Lamberti et al., 2011b). Here, we examined the effects of SSU peptide fragment containing a phosphorylation site on the mitochondrial import of pF1β. First, RRL-pF1β-1 was mixed with a peptide derived from tobacco pSSU (28-37; GLKSAASFPV) which contains the phosphorylatable Ser-34 site during *in vitro* phosphorylation step prior to mitochondrial import. Before performing mitochondrial import assay for 5 min, RRL-pF1β-1 was incubated with or without 5 μg LE in the presence of pSSU peptides at 26°C for 2, 4 and 6 min. As shown in **Figure 6A**, LE treatment impeded the import of the RRL-pF1β-1 (lanes 3 and 6), but this was partially relieved by the inclusion of SSU peptide (lanes 4 and 7). However, the import rates of LE-treated RRL-pF1β-1 (lanes 9 and 10) with or without pSSU peptides were indifferent when the phosphorylation period was extended to 6 min, suggesting that the majority of pSSU peptides have been phosphorylated at 6 min and lost the ability to compete for LE kinases.

To obtain further evidence to show that there was a competition for LE kinases between RRL-synthesized pF1βs and pSSU peptides, a non-phosphorylatable mutant pSSU-M31/34-S/A peptide (28-37; GLKAAAAFPV) containing two serine to alanine mutations (M) was used in this study (May and Soll, 2000). As shown in **Figure 6B**, the import of LE-treated RRL-pF1β-1/2/3 were enhanced when phosphorylatable pSSU peptide (lane 4) was present, whereas the pSSU-M31/34-S/A peptide had no effect (lane 5). These data suggested that there was a competition for LE kinases between RRL-synthesized pF1βs and pSSU peptides.

## Discussion

During the evolution of endosymbionts, the genomes of the ancient prokaryotic ancestors of chloroplasts and mitochondria have been greatly reduced. It is believed that many genes with no contribution to endosymbiosis have been discarded and thousands of functional genes beneficial to endosymbiosis have been transferred to the nucleus of plant cells. After transcription in the nucleus, their mRNAs are translated into proteins in cytosol which are then imported into chloroplasts and/or mitochondria through the main entry gates on the outer membranes, TOC and TOM complexes, respectively (Schleiff and Becker, 2011; Jarvis and López-Juez, 2013). Since both organelles have evolved from prokaryotic origins, it is not surprising that some of their import mechanisms might be alike. Most of the nuclear-encoded preproteins are sorted to the correct destinations based on their N-terminal sequences (transit peptides of chloroplast proteins or presequences of mitochondrial proteins) (Schleiff and Becker, 2011; Chotewutmontri et al., 2012). The import mechanism of nuclear-encoded chloroplast preproteins has been comparatively well-studied in the last two decades. Before binding to the TOC complex, the transit peptides of some nuclear-encoded chloroplast preproteins are phosphorylated by STY dual-specificity kinases and form a guidance complex with 14-3-3 protein and cytosolic Hsp70 (May and Soll, 2000; Martin et al., 2006; Lamberti et al., 2011a). In our recent study, we showed that phosphorylation and dephosphorylation of the presequence of pMORF3 are involved in its import into mitochondria (Law et al., 2015). We found that the presequences of pMORF3/5/6 contain phosphorylation sites, which could be phosphorylated by STY8 and STY17 kinases. In addition, we demonstrated that phosphorylated pMORF3 forms a complex with 14-3-3 protein and cytosolic Hsp70 (Law et al., 2015). These findings raise a question: Are phosphorylation of transit peptides and presequences by cytosolic kinases a common regulatory mechanism to the import of certain nuclear-encoded proteins into these two organelles?

Using data-mining approach from the PhosPhAt 4.0 Database (Zulawski et al., 2013), 103 mitochondrial proteins were found to contain experimentally proven phosphorylation sites on their N-terminal sequences (Supplementary Table S1). The N-terminal sequences of several representative nuclear-encoded mitochondrial preproteins were chosen as substrates for *in vitro* kinase assays to verify the mass spectrometry data (**Figures 2, 3**). Our data showed that phosphorylation of N-terminal sequences is common to some of the nuclear-encoded mitochondrial preproteins (**Figures 2, 3**). More importantly, different kinases have different substrate specificities. For example, some sequences can be phosphorylated by STY8 kinase but not by STY17 kinase (**Figure 2**). The three STY kinases are not the only kinases that can phosphorylate presequences in Arabidopsis. The N-terminal sequences of two homologs of Arabidopsis pF1β-1/3 are not phosphorylated by STY kinases but are phosphorylated by unknown kinases in LE and RE (**Figure 3**). The uncharacterized pF1β-phosphorylating kinases in RE and LE could be different kinases with different specificities, as the presequence of pMORF3 can be phosphorylated by LE but not by RE (**Figure 3**). In previous studies, it was reported that Arabidopsis LE and STY kinases cannot phosphorylate pF1β from *Nicotiana plumbaginifolia* (Waegemann and Soll, 1996; Martin et al., 2006). This may be because the enzyme (*A. thaliana*) and substrate (*N. plumbaginifolia*) were from different species. To date, STY kinases are the only known kinase family which could phosphorylate both well-studied nuclear-encoded chloroplast preprotein (pSSU) (Martin et al., 2006) and mitochondrial preprotein (pMORF3) (Law et al., 2015). Meanwhile, a dual-targeted mitochondrial and chloroplast outer membranes phosphatase, AtPAP2, could interact with both nuclear-encoded mitochondrial and chloroplast preproteins and may play a role in dephosphorylating phosphorylated transit peptides and presequences (Law et al., 2015; Zhang et al., 2016).

The import rate of preproteins can be regulated by phosphorylation of their transit peptides (pSSU) or presequences (pMORF3) through formation of a guidance complex with 14-3-3 and Hsp70 (May and Soll, 2000; Lamberti et al., 2011a; Law et al., 2015). Complex formation of pSSU/14-3-3/Hsp70 was shown to enhance the import efficiency of pSSU into chloroplasts (May and Soll, 2000), but complex formation of pMORF3/14-3-3/Hsp70 impedes the import efficiency of pMORF3 into the mitochondria (Law et al., 2015). In contrast, monomeric form of phosphorylation-deficient mutant of pMORF3 displayed faster rates of import (Law et al., 2015). As shown in **Figure 6**, the phosphorylatable pSSU peptide but not the non-phosphorylatable pSSU peptide could enhance import of LE-treated RRL-pF1β-1/2/3 into mitochondria, suggesting that there was a competition for LE kinases between RRL-synthesized pF1βs and pSSU peptides. Therefore, phosphorylation of transit peptides/presequences, an energy-consuming process, may serve a biological purpose in regulating and coordinating protein import into these two organelles. The presence of multiple kinases with different substrate specificities may allow selective phosphorylation under various physiological conditions. The kinases in different tissues (e.g., leaf and root) could therefore phosphorylate different preproteins and control the priority of protein import into plastids/mitochondria in different tissues (Zulawski et al., 2013). There are three homologous pF1β genes in Arabidopsis genome. While their mature sequences (a.a. 70–end) are 100% conserved in a.a. sequence identity (Supplementary Figure S1), their sequence variations only happen at the N-terminal sequences. The difference in N-terminal sequences may enable differential import regulation of pF1βs by differential phosphorylation by different kinases in various tissues or developmental stages. For example, STY46 can phosphorylate the N-terminal sequence of pF1β-2 but not the N-terminal sequences of pF1β-1/3 (**Figure 2**), but the N-terminal sequence of pF1β-2 was less intensely phosphorylated by LE and RE kinases than the N-terminal sequences of pF1β-1/3 (**Figure 3**). The T-DNA lines of STY kinases exhibited different growth phenotypes, indicating that they cannot completely complement each other (Lamberti et al., 2011b). This could be due to their different substrate specificity.

After phosphorylation by LE, the N-terminal sequences of pF1βs can bind to 14-3-3 (**Figure 4**). The binding of phosphorylated transit peptides/presequences to 14-3-3 protein may offer an additional regulatory mechanism. There are thirteen 14-3-3 genes in the Arabidopsis genome (TAIR). Pull-down assays and mass spectrometry showed that 14-3-3 isoforms chi (χ) and epsilon (𝜀) bind differentially to different but overlapped client proteins from developing Arabidopsis seeds (Swatek et al., 2011). While it is not known how many 14-3-3 isoforms could recognize phosphorylated transit peptides and/or presequences of preproteins, it is possible that the same phosphorylated transit peptide/presequence can be recognized and regulated by different 14-3-3 isoforms. One example is pF1β-1, which was pulled down by 14-3-3 isoforms χ and 𝜀 from developing seed extracts (Swatek et al., 2011). Hence, it would not be surprising if the thirteen 14-3-3 isoforms may have different binding specificities to various phosphorylated transit peptides/presequences and form complexes with different 14-3-3 isoforms. It is also possible that complexes with different 14-3-3 isoforms may preferentially interact with different TOC and TOM receptors. This may explain why two isoforms of Toc33/34 and four isoforms of Tom20 receptors are maintained in the Arabidopsis genome (Schleiff and Becker, 2011).

In animal system, a cytosolic protein, mitochondrial import stimulating factor (MSF), was shown to bind to the presequences of some mitochondrial proteins (Hachiya et al., 1993, 1994). MSF was later shown to be a 14-3-3 protein (Alam et al., 1994). After the docking of the MSF-precursor protein complex to Tom37/Tom70, the MSF is released from the precursor in an ATP-dependent manner. The precursor is then transferred to Tom20/Tom22 and imported into mitochondria (Komiya et al., 1998). It was shown that RRL-translated animal precursor proteins were import-competent to rat mitochondria, but that of WGL-translated precursor proteins were import-incompetent. Interestingly, addition of MSF can prevent aggregation of the WGL-translated adrenodoxin precursor and stimulate its import into rat mitochondria. Hence, the binding of 14-3-3 to presequences seems to be common in both animal and plant systems, although the outcomes are opposite. Binding of MSF to the presequence of adrenodoxin enhances its import into rat mitochondria, and binding of 14-3-3 to the transit peptide of pSSU also enhances its import into chloroplasts (May and Soll, 2000). By contrast, binding of 14-3-3 to the presequences of pMORF3 and pF1β impedes their import to plant mitochondria. This differential mechanism could be specially evolved in plants as plant cells contain both plastids and mitochondria.

MSF was shown to bind to the arginine-rich presequence of adrenodoxin precursor and prevent its aggregation. However, this ability was abolished when two arginine residues on the presequence were mutated to serine (Hachiya et al., 1993). Interestingly, the N-terminal sequences of nuclear-encoded mitochondrial proteins of *H. sapiens* (mean charge of *N*-termini is 3.1) are rich in arginine but not serine (R > S), and those present in *S. cerevisiae* (mean charge of N-termini is 3.4) are rich in both arginine and serine residues (R = S) (Garg and Gould, 2016). In *A. thaliana*, the N-terminal sequences of nuclear-encoded mitochondrial proteins are generally strong in positive charges (mean charge of *N*-termini is 3.4) and rich in arginine and serine residues (R = S), whereas the transit peptides of nuclear-encoded chloroplast proteins are less positively charged (mean charge of *N*-termini is 1.0) and rich in serine but not in arginine (S > T > R). In our study, serine and threonine are the major experimentally proven phosphorylated residues in Arabidopsis N-terminal sequences (Supplementary Table S1). Of the 104 experimentally proven phosphorylated mitochondrial N-terminal sequences, 85 serine and 42 threonine residues were phosphorylated. Phosphorylation of these residues will reduce the mean charge of N-termini. The low incidence of serine residues on the N-terminal sequences of nuclear-encoded mitochondrial proteins of *H. sapiens* implies that phosphorylation and dephosphorylation of presequences are less likely to be present in animals, as homologs of STY kinases and AtPAP2 can only be found in the genomes of green algae and higher plants. These may be substantiated by the facts that the kinase activities of RRL was much lower than WGL (Law et al., 2015), and the binding of MSF to the arginine-rich presequence of adrenodoxin precursor is not dependent on kinase activity (Hachiya et al., 1993).

**FIGURE 7:**
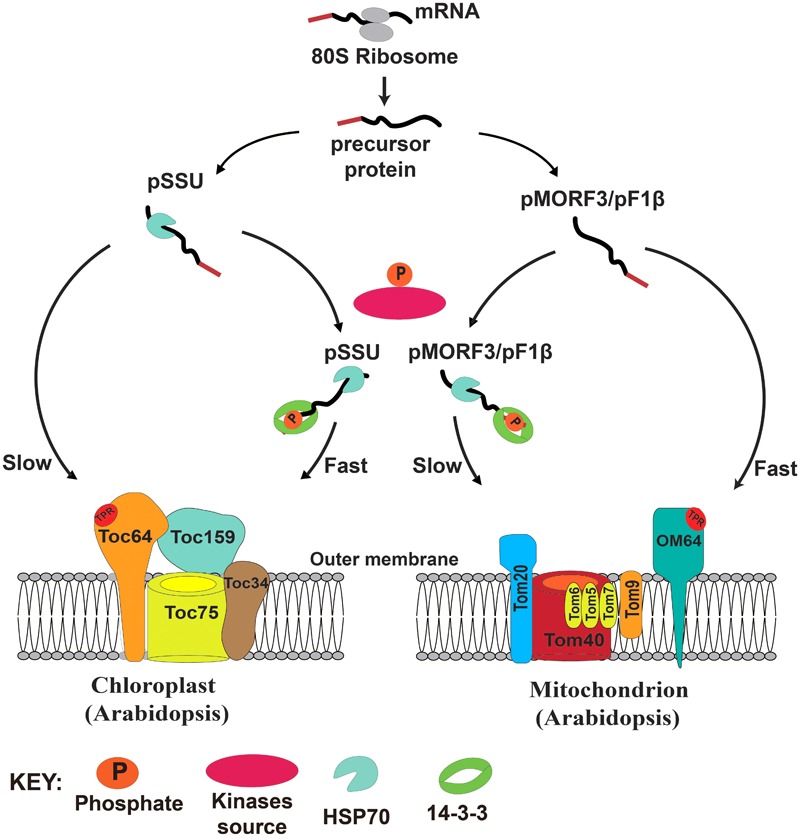
A model of the regulatory roles of phosphorylation of transit peptides/presequences on chloroplast and mitochondrial import.

In yeast, more than 30 phosphorylation sites have been identified on the TOM complex (Gerbeth et al., 2013a). Phosphorylation of TOM components by casein kinase 2 (CK2) can promote TOM biogenesis (Schmidt et al., 2011), whereas phosphorylation of Ser54 of Tom40 and Thr76 of Tom 22 by protein kinase A (PKA) impedes their assembly to the TOM complex (Rao et al., 2012; Gerbeth et al., 2013b). Phosphorylation of Ser174 of Tom70 by PKA can also reduce import of metabolite precursors (Schmidt et al., 2011). It is proposed that switching from respiration to fermentation by addition of sugars activates cAMP and thus PKA activity, which in turn reduces energy production capacity of mitochondria by suppressing protein import. Here, we propose that the presence of multiple transit peptide/presequence-phosphorylating kinases with distinct but overlapping substrate specificities might prioritize the import of proteins into these two energy organelles in plants. In our model, newly translated preproteins from cytosolic ribosomes either remain in a free non-phosphorylated (monomeric) form or become phosphorylated by kinase sources in the cytosol. The phosphorylated pSSU can form a complex with 14-3-3 and cytosolic Hsp70, and the import rate of this complex into chloroplasts is faster than that of the monomeric pSSU. By contrast, the mitochondrial import rate of the phosphorylated pMORF3/pF1βs, complexed with 14-3-3 and Hsp70, is slower than that of the monomeric unphosphorylated pMORF3/pF1βs (**Figure 7**). Phosphorylation of presequences has yet to be shown to affect protein import into mitochondria of animal or yeast. This design may have evolved in plants to coordinate protein import into chloroplasts and mitochondria.

## Accession Numbers

Sequence data from this work can be found in the Arabidopsis Genome Initiative or GenBank/EMBL databases under the following accession numbers: AtCytC1, At5g40810; ATP17, At4G30010; AtPAP2, At1g13900; CAL2, At3g48680; COX5b1, At3g15640; F1β-1, At5g08670; F1β-2, At5g08680; F1β-3, At5g08690. MORF3, At3g06790; SDH1-2, At2g18450; SSU, At5g38430; STY8, At2g17700; STY17, At4g35708, and STY46, At4g38470.

## Author Contributions

BL designed the study. LN and JY produced overexpression constructs. SC carried out phylogenetic analysis. LK and YS carried out data-mining of experimentally proven phosphorylation sites and putative 14-3-3 binding sites on N-terminal sequences. Y-SL carried out all the other experiments and wrote the manuscript. SS provided guidance on this study, and all authors were involved in writing and/or revising the manuscript.

## Conflict of Interest Statement

The authors declare that the research was conducted in the absence of any commercial or financial relationships that could be construed as a potential conflict of interest.

## Footnotes

1 http://www.compbio.dundee.ac.uk/1433pred/

2 http://weblogo.berkeley.edu/logo.cgi

## Abbreviations

ATP17: ATP synthase 17
CAL2: gamma carbonic anhydrase-like 2
COX5b1: cytochrome *c* oxidase subunit 5b-1
CytC1: cytochrome C1
F1β: mitochondrial ATP synthase subunit beta 1
Hsp70: heat shock protein 70
LE: leaf extract
MORF3: multiple organellar RNA editing factors 3
pSSU: precursor of small subunit of Rubisco
RE: root extract
RRL: rabbit reticulocyte translation lysate
SDH1-2: succinate dehydrogenase 1-2
STY kinases: Ser/Thr/Tyr kinases
TOC complex: translocases of the outer chloroplast
TOM complex: translocases of the outer mitochondrial
WGL: wheat germ lysate
