# Supplementary material for: Multiple Kinases Can Phosphorylate the N-Terminal Sequences of Mitochondrial Proteins in Arabidopsis thaliana
Source: Front Plant Sci. 2018 Jul 10;9:982. doi: 10.3389/fpls.2018.00982 (PMC6048449; doi:10.3389/fpls.2018.00982)
Supplement: Supplementary file 2 [file Image_1.PDF]

SUPPLEMENTARY FIGURE

```
                             10        20        30        40        50
                     ....|....|....|....|....|....|....|....|....|....|
AtF1β-1              MASRRVLSSLLRSSSGRSAAK---LGNRNPRLPSPSPARPAAPCSYLLGR
AtF1β-3              MASRRVLSSLLRSSSGRSAAK---LVNRNPRLPSPSPARHAAPCSYLLGR
AtF1β-2              MASRRILSSLLRSSSSRSTSKSSLIGSRNPRLLSPGPAHGAAPCGTLLGR
Clustal Consensus    *****:*********.**::*   : .***** **.**: ****. ****

                             60        70        80        90       100
                     ....|....|....|....|....|....|....|....|....|....|
AtF1β-1              VAEYATSSPASSAAPSSAPAKDEGKKTYDYGGKGAIGRVCQVIGAIVDVR
AtF1β-3              VAEYATSSPASSAAPSSAPAKDEGKKTYDYGGKGAIGRVCQVIGAIVDVR
AtF1β-2              VAEYSTSSPANSAAPSSAPAKDEGKKTYDYGGKGAIGRVCQVIGAIVDVR
Clustal Consensus    ****:*****.***************************************

                            110       120       130       140       150
                     ....|....|....|....|....|....|....|....|....|....|
AtF1β-1              FEDQEGLPPIMTSLEVQDHPTRLVLEVSHHLGQNVVRTIAMDGTEGLVRG
AtF1β-3              FEDQEGLPPIMTSLEVQDHPTRLVLEVSHHLGQNVVRTIAMDGTEGLVRG
AtF1β-2              FEDQEGLPPIMTSLEVQDHPTRLVLEVSHHLGQNVVRTIAMDGTEGLVRG
Clustal Consensus    **************************************************

                            160       170       180       190       200
                     ....|....|....|....|....|....|....|....|....|....|
AtF1β-1              RKVLNTGAPITVPVGRATLGRIMNVLGEPIDERGEIKTEHYLPIHRDAPA
AtF1β-3              RKVLNTGAPITVPVGRATLGRIMNVLGEPIDERGEIKTEHYLPIHRDAPA
AtF1β-2              RKVLNTGAPITVPVGRATLGRIMNVLGEPIDERGEIKTEHYLPIHRDAPA
Clustal Consensus    **************************************************


                     ...
AtF1β-1              LVD
AtF1β-3              LVD
AtF1β-2              ---
Clustal Consensus
```

Supplementary Figure S1. Multiple a.a. sequence alignment of F1β-1/2/3 and NpF1β. Boxes indicate experimentally-proven phosphorylation sites.
